# Gene expression profiling of liver metastases from colorectal cancer as potential basis for treatment choice

**DOI:** 10.1038/sj.bjc.6604681

**Published:** 2008-09-30

**Authors:** M A Pantaleo, A Astolfi, M Nannini, P Paterini, G Piazzi, G Ercolani, G Brandi, G Martinelli, A Pession, A D Pinna, G Biasco

**Affiliations:** 1 Institute of Hematology and Medical Oncology ‘L. A. Seragnoli’, S.Orsola-Malpighi Hospital, University of Bologna, Bologna, Italy; 2 Pediatric Oncology and Hematology, University of Bologna, Bologna, Italy; 3 Centre of Applied Biomedical Research (CRBA), S.Orsola-Malpighi Hospital, University of Bologna, Bologna, Italy; 4 Liver and Multi-organ Transplantation Unit, University of Bologna, Bologna, Italy

**Keywords:** gene expression profiling, microarrays, colorectal cancer, liver metastases, epidermal growth factor receptor, cyclooxygenase-2

## Abstract

At present no reports on gene expression profiling of liver metastases from colorectal cancer are available. We identified two different signatures using Affymetrix platform: epidermal growth factor receptor pathway was upregulated in metachronous lesions, whereas the pathway mainly related to angiogenesis was in synchronous lesions. Synchronous or metachronous liver metastases could be treated differently on the basis of different molecular pathways.

The liver is the most common site of metastases from colorectal cancer. About 25% of patients presented liver metastases at diagnosis and about 70% of patients develop a liver recurrence after radical surgery of colorectal tumours (50% of patients with stage III and 20% with stage II cancer) (Penna and Nordlinger, 2002). The treatment of metastases from colorectal cancer is complicated and still controversial (Biasco *et al*, 2006; Ercolani *et al*, 2006). For unresectable lesions, the medical systemic treatment is considered the standard option. During the last few years, novel biological agents such as monoclonal antibodies inhibiting growth factor receptors or angiogenesis have been combined with chemotherapy to improve the outcome of patients affected by colorectal cancer (Cunningham *et al*, 2004; Hurwitz *et al*, 2004; Saltz *et al*, 2004, 2007; Giantonio *et al*, 2007; Tabernero *et al*, 2007; Van Cutsem *et al*, 2007). In clinical practice the choice of treatments are based only on published clinical data with the respect to first-, second- and third-line therapy. However, the natural history, the clinical scenario and the prognosis of liver metastases may be different. From a general clinical point of view, liver metastases are classified as ‘synchronous’ lesions if they are present at diagnosis of disease or if they occur less than 6 months after surgery of primary tumour and ‘metachronous’ lesions if they occur after more than 6 months. At present, the systemic therapy is not differentiated for these two clinical settings. The aim of this study is to study the gene expression profiling of synchronous and metachronous liver metastases using Affymetrix platform to identify molecular patterns as a possible basis for the choice of systemic therapies and for response prediction.

## Materials and methods

### Patients and tissues

This study was approved by the local Ethical Committee (approval number: 6/2005/U/Tess). Fresh tissue specimens from liver metastases of 18 patients who had undergone liver surgery were collected after written consent. The specimens were obtained from a single lesion for each patient to avoid the inter-lesion biological variability and immediately frozen in the operating room in liquid nitrogen. The lesions were classified as 10 synchronous and 8 metachronous lesions. The patient's characteristics are described in Table 1.

### Microarray analysis

Total RNA was extracted from frozen tumour specimens using TRIzol Reagent (Invitrogen Life Technologies, Carlsbad, CA, USA), labelled and hybridised to HG-U133Plus 2.0 Affymetrix arrays following the manufacturer's instructions. Data shown in this publication have been deposited in the NCBI Gene Expression Omnibus database. Raw data were background-subtracted, normalised and summarised with the robust multi-array average (RMA) algorithm implemented in the *affy* package of Bioconductor (http://www.bioconductor.org). Routine quality controls available in the *affy* and *affyPLM* packages of Bioconductor were performed to check for the presence of artifacts and for the consistency of normalisation across arrays. Probes poorly expressed in more than 8 samples out of 18 or not changing among the samples, based on interquartile range (IQR) calculation, were excluded from further analysis. Genes differentially expressed between synchronous and metachronous lesions were selected by the permutation-based *t*-statistics implemented in the SAM algorithm (Tusher *et al*, 2001). SAM computes the false discovery rate (FDR, the proportion of false positives in output list of differential genes) by permutations of the sample labels. We set the FDR threshold for significance at 5%. All the analyses were performed with R 2.6.0 and Bioconductor packages. Heatmap representation of differentially expressed genes was performed with MeV software (http://www.tm4.org/mev.html), and pathway analysis with EASE tool (http://david.abcc.ncifcrf.gov/), calculating the significance of enrichment of a pathway by the EASE score.

Following the suggestions from the microarrays analysis and in order to confirm the data, quantitative determinations were performed of most clinical relevant proteins such as cyclooxygenase-2 (COX-2) and epidermal growth factor receptor (EGFr).

### Real-time PCR quantification of COX-2 and EGFr

Total RNA was reverse transcribed using Superscript II (Invitrogen Life Technologies) with oligo-dT primers, according to the manufacturer's guidelines. Gene-specific primers and TaqMan probes were designed with the Beacon Designer 2.0 Software (Premier Biosoft International, Palo Alto, CA, USA) and real-time PCR was performed using an iCycler apparatus (Bio-Rad Laboratories, Hercules, CA, USA). The cycle numbers were recorded when the accumulated PCR products crossed an arbitrary threshold (CT or threshold cycle) and CT values were used to calculate the expression levels of COX-2 and EGFr relative to the average of two housekeeping genes *β*-actin and 18S rRNA.

### Protein extraction and COX-2 western blot analysis

Frozen tissues were homogenised using lysis buffer (50 mM Tris pH 7.4, 150 mM NaCl, 2 mM MgCl2, 1% Triton X-100, 10% glycerol, 2 mM EGTA, 1 mM DTT) containing protease inhibitors (10 mg ml^−1^ aprotinin and leupeptin, 5 mg ml^−1^ pepstatin, 1 mM PMSF) and phosphatase inhibitors (50 mM NaF, 10 mM Na_4_P_2_O_7_, 1 mM Na_3_VO_4_, 3 mM H_2_O_2_). Samples were processed according to the standard procedures: anti COX-2 antibody (BD Transduction Laboratories, Lexington, KY, USA), incubations conditions: 1 : 500 in TBS-TB buffer (50 mM Tris-HCl, pH 7.4, 150 mM NaCl, 1% Tween 20 and 3% bovine serum albumin) at 4°C o.n.

### ELISA quantification of EGFr

The concentration of total EGFr was assessed using the ELISA kits purchased from Biosource International Inc. (Camarillo, CA, USA). Protein lysates from A431 and SW620 cell lines were used respectively as positive and negative controls, to verify the specificity of the EGFr ELISA assays. Protein quantification was expressed using box plots. Significance was analysed by non-parametric log-rank test (Mann–Whitney test). A *P*-value less than 0.05 was considered significant. The statistical calculations were performed using StatView 5.0 statistical software (SAS Institute Inc., Cary NC, USA). (Details on Materials and Methods are available in Supplementary Information).

## Results

**Figure 1:**
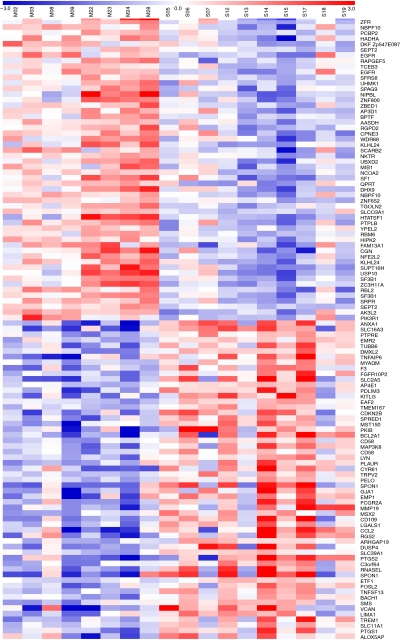
Heatmap representation of genes differentially expressed between metachronous (M) and synchronous (S) liver metastases: in blue (underexpressed genes, log2 ratio=−3) and in red (overexpressed genes, log2 ratio=3) representing the two extremeties of gene expression. Log ratios are referred to average expression level in all samples for each gene.

**Figure 2:**
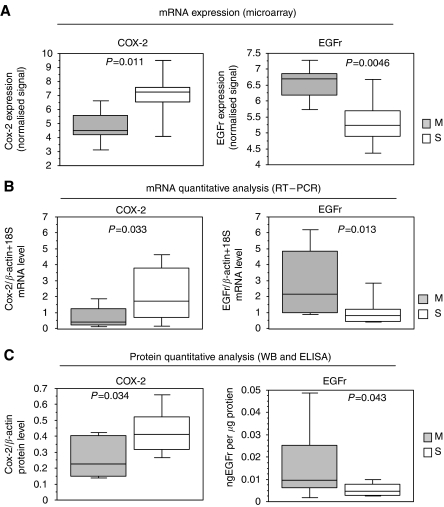
COX-2 and EGFr differences in synchronous and metachronous metastases. (**A**) mRNA expression analysed by microarray and shown as normalised expression value as calculated by RMA algorithm; (**B**) mRNA expression with real-time PCR analysis; (**C**) protein quantification with western blotting for COX-2 and ELISA for EGFr.

The gene expression analysis identified 49 genes upregulated in metachronous and 55 genes upregulated in synchronous metastases with a FDR <5% (Figure 1). Among these, functional analysis of differential genes showed two main deregulated pathways of clinical interest in medical oncology: EGFr signalling pathway (*P*=0.065, modified Fisher's exact test) and eicosanoid metabolism (*P*=0.012). Key genes belonging to these pathways are EGFr, PIK3R1, the regulatory subunit 1 (p85-*α*) of the phosphoinositide-3-kinase, COX-2, COX1 and ALOX5AP, the activating protein of the arachidonate 5-lipoxygenase. In particular, EGFr was overexpressed in metachronous lesions (*P*=0.046, Mann–Whitney test) and COX-2 gene was overexpressed in synchronous lesions (*P*=0.012) (Figure 2A). To confirm the differential expression of these two genes, a quantitative analysis of EGFR and COX-2 mRNA with real-time PCR, and of protein levels by western blot (COX-2) and ELISA test (EGFr) were performed. Both analyses showed that COX-2 was overexpressed in synchronous lesions (*P*=0.033 and *P*=0.034) and EGFr was overexpressed in metachronous lesions (*P*=0.013 and *P*=0.043), respectively, at the mRNA and protein levels (Figure 2B and C).

## Discussion

Over the few last years, the gene expression profiling analysis with microarray technology has shown a great potential for clinical application in medical oncology (Bittner *et al*, 2000; Perou *et al*, 2000; Dhanasekaran *et al*, 2001; Garber *et al*, 2001; Takahashi *et al*, 2001; van ‘t Veer *et al*, 2002; Van de Vijver *et al*, 2002; Ayers *et al*, 2004; Jones *et al*, 2005; Dressman *et al*, 2006; Bonnefoi *et al*, 2007; Bueno-de-Mesquita *et al*, 2007; Kim *et al*, 2007). Several data have already been published on the powerful prognostic role of the gene signature in many tumours (Bittner *et al*, 2000; Dhanasekaran *et al*, 2001; Garber *et al*, 2001; Takahashi *et al*, 2001; van ‘t Veer *et al*, 2002; Van de Vijver *et al*, 2002; Jones *et al*, 2005; Bueno-de-Mesquita *et al*, 2007) and also on the predictor role of complete response to neoadjuvant chemotherapy especially in breast cancer (Ayers *et al*, 2004; Dressman *et al*, 2006; Bonnefoi *et al*, 2007; Kim *et al*, 2007). Concerning colorectal cancer, several reports are available but they are mostly aimed at improving the diagnosis on a molecular basis differentiating between cancer, adenoma and normal mucosa, and also to evaluating the potential for metastases developing (Alon *et al*, 1999; Notterman *et al*, 2001; Yanagawa *et al*, 2001; Bertucci *et al*, 2004; Koehler *et al*, 2004; Li *et al*, 2004; Wang *et al*, 2004; Eschrich *et al*, 2005). Moreover, the studies have been mainly conducted on primary tumours and only very few data are available on metastases (Yanagawa *et al*, 2001; Koehler *et al*, 2004).

This study first reports the gene expression profiling of liver metastases from colorectal cancer. Our results showed that the molecular background of liver metastases may be different and that EGFR and COX-2 are overexpressed in metachronous and synchronous metastases, respectively. These findings improve the current knowledge on biological background of colorectal liver metastases. Furthermore, they may also have some clinical implications, because they suggest that medical treatments of patients with liver metastases may be differentiated in these two clinical setting according to their different biological background. Therapies based on EGFr pathway inhibition may be considered for metachronous metastases (such as monoclonal antibodies cetuximab or panitumumab) and therapies based on angiogenesis cross-talking pathways inhibition (such as the monoclonal antibody bevacizumab, COX-2 inhibitors or small molecules tyrosin kinase inhibitors with antiangiogenetic properties) for the synchronous metastases.

Regarding EGFr pathway in colorectal cancer, at present, no molecular factors are predictive of response to cetuximab or panitumumab-based treatments except for K-ras mutations (De Roock *et al*, 2008; Lièvre *et al*, 2008). In fact, it is already well known that morphological expressions of the receptor studied with immunohistochemistry is inadequate and other evaluations did not reach any conclusive data (Dei Tos and Ellis, 2005; Moroni *et al*, 2005; Vallböhmer *et al*, 2005; Lenz *et al*, 2006). In our study, the expression of EGFr has been evaluated using three different molecular techniques that provide quantitative information that, in our opinion, may be a more accurate and reliable study of EGFr status in colorectal cancer as a predictor of response to EGFr inhibitors.

The COX-2 upregulation in synchronous metastases supports its association with tumour invasiveness and metastatic process because COX-2 affects cell proliferation, tumour growth, angiogenesis, apoptosis resistance and immune response (Sheng *et al*, 1997; Chen *et al*, 2001). COX-2 overexpression may suggest a more aggressive phenotype of this kind of metastases that require a treatment preferentially directed against tumour angiogenesis, such as bevacizumab-based combinations or a treatment creating an unfavourable environment for tumour growth as recently published with COX-2 inhibitors (de Heer *et al*, 2008).

As future perspective, from a biological point of view, it could be interesting to compare gene expression profiling of metastases and primary tumour to better understand the molecular mechanisms involved in the metastatic process and to early identify main predicting genes of metachronous metastases development.

In conclusion, synchronous and metachronous liver metastases from colorectal cancer have a different gene expression signature and a different expression of EGFR and COX-2 that may be the basis for choosing the medical treatment. These preliminary results need to be confirmed in larger series and, in the future, their role as molecular predictors should be also investigated in clinical trials.

## Figures and Tables

**Table 1:** Patient's and tumours characteristics

**Number of patients**	**Total 18**
*Sex*	
Male	10 (66.6%)
Female	8 (33.3%)
	
*Age*	
Median	63 years
Range	41–77 years
	
*Primary tumour site*	
Right colon	6 (33.3%)
Left colon	8 (44.4%)
Rectum	4 (22.2%)
	
*Synchronous/metachronous*	
Synchronous	10 (55.5%)
Metachronous	8 (44.4%)
	
*Single/multiple metastases*	
Single	4 (22.2%)
Multiple	14 (77.7%)
