# Supplementary material for: Gene expression profiling of liver metastases from colorectal cancer as potential basis for treatment choice
Source: Br J Cancer. 2008 Sep 30;99(10):1729–34. doi: 10.1038/sj.bjc.6604681 (PMC2584956; doi:10.1038/sj.bjc.6604681)

**Supplementary material**

**Materials and Methods**

**Liver metastases analysis.** Approximately 30 mg of liver metastasis from colorectal cancer tissue was pulverized with mortar an pestle while frozen in liquid nitrogen. TriZol reagent [Invitrogen Life Technologies, Carlsbad, CA, USA] was added and total RNA was extracted from tumours following the manufacturer’s protocol. RNA was quantified with a Nanodrop ND-1000 spectrophotometer [Nanodrop, Thermo Fisher Scientific, Wilmington, Delaware USA] and quality was checked by ethidium bromide stained gel electrophoresis. Only samples with a 260/280 absorbance ratio >1.8 and with no evidence of smears of ribosomal RNA bands were used for analyses.

**Gene expression arrays.** Hybridization targets (samples for hybridization) were prepared from total RNA according to standard Affymetrix protocols. The amount of starting total RNA for each reaction was 5 μg. Briefly, first-strand cDNA was generated using a T7- linked oligo-dT primer, followed by second-strand synthesis. An in vitro transcription reaction was performed to generate cRNA containing biotinylated UTP and CTP, which was then chemically fragmented at 95°C for 35 min. 15 μg of fragmented, biotinylated cRNA was incubated in hybridization buffer, denatured and hybridized to the Affymetrix GeneChip Human U133 Plus 2.0 arrays at 45°C for 16 hr, according to the directions of the manufacturer. The arrays contain over 54,000 probes, representing more than 47000 human transcripts. Arrays were then washed and stained with streptavidin-phycoerythrin [SAPE, Molecular Probes, Invitrogen]. Signal amplification was performed using a biotinylated anti-streptavidin antibody [Vector Laboratories, Burlingame, CA] and goat IgG [Sigma-Aldrich]. Scans were performed with an Affymetrix GeneChip scanner 7G. Samples with a percentage of genes called Present by the MAS5 Detection algorithm < 40% were excluded from the analysis.

**Microarrays analysis**

The expression value for each gene was calculated using the robust multi-array average (RMA) algorithm implemented in the Bioconductor extensions (http://www.bioconductor.org) to the R statistical environment. RMA generates log-2 scaled measures of expression on quantile-normalized probe-level expression data. Probes with a small variation between samples, calculated as the inter-quartile range of expression level, and with low expression level in more than 8 samples out of 18 were removed.

**Statistical analysis**. We carried out the statistical analysis of differential gene expression using the SAM algorithm (Tusher *et al*, PNAS, 98:5116-21, 2001) that estimates false discovery rate by permutation of the sample labels. SAM computes a statistic d_i_ for each gene *i,* measuring the strength of the relationship between gene expression and the response variable. The *d*-statistic a variant of the *t*-statistic that uses a fudge factor *s_0_* (a small positive constant) to limit the effect of high variation at low intensities. SAM uses repeated permutations of the data to determine if the expression of any genes are significantly related to the response. The cut-off for significance is determined by a tuning parameter delta, chosen by the user based on the false positive rate. We set the false discovery rate (FDR) threshold to 5%, meaning that we accepted 5% false positives in the output list of differential genes. Differential genes were shown by a heatmap representation. EGFR and COX-2 expression levels in microarray analysis were also represented by a boxplot for comparison to real-time PCR and protein level analyses. Statistical significance was calculated by Mann-Whitney U test.

Pathway analysis was performed by the DAVID/EASE tool (<http://david.abcc.ncifcrf.gov/>) calculating the significance of enrichment of a pathway by the EASE score, a modified Fisher Exact p-value. This test calculates the probability to find a given biological theme or pathway based on the list of genes differentially expresses by chance alone. The EASE score penalizes the significance of categories supported by fewer genes by a procedure called jackknifing, in which a single data point is removed and the statistic is recalculated many times to give a distribution of probabilities that is broad if the result is highly variable. Fisher Exact P-Value and EASE score range from 0 to 1, in which 0 represents perfect enrichment. Default cut-off is 0.1.

**Results**

The expression of COX-2 in the different samples is shown on the following figure:


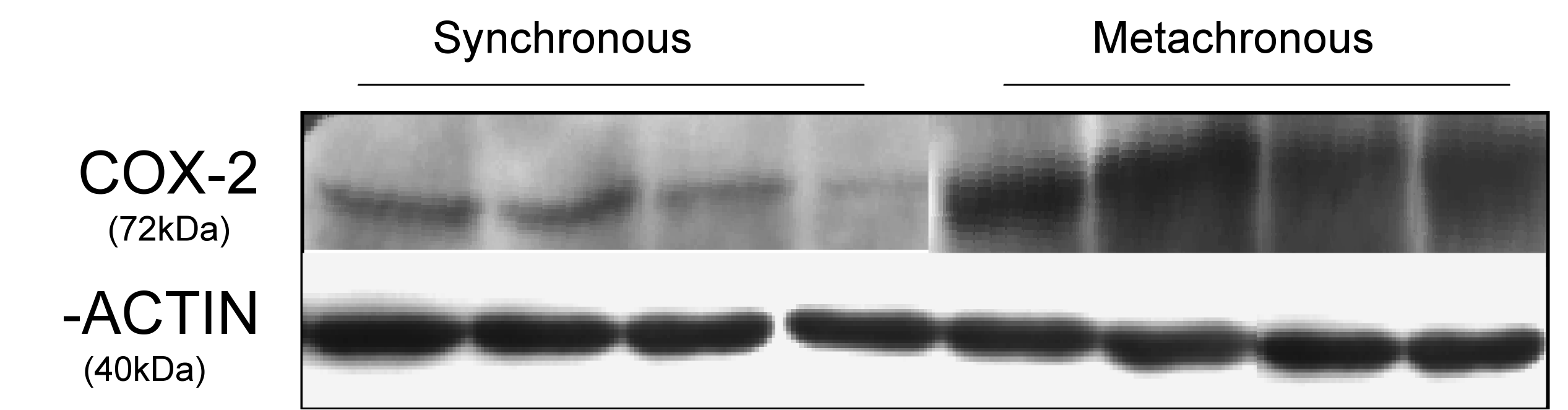

Supplement: Supplementary Information [file 6604681x1.doc]
